# Seven New Species of *Anastatus* Motschulsky (Hymenoptera: Chalcidoidea: Eupelmidae) from China Identified Based on Morphological and Molecular Data

**DOI:** 10.3390/insects15110893

**Published:** 2024-11-15

**Authors:** Zongying Wang, Yihang Zhou, Yaxuan Zou, Qifei Liu, Lingfei Peng

**Affiliations:** 1 Biological Control Research Institute, Fujian Agriculture and Forestry University, Fuzhou 350002, China; wangzy902@163.com (Z.W.); zyh6453088@163.com (Y.Z.); 15959195960@163.com (Y.Z.); 2 UN (China) Center for Fruit Fly Prevention and Treatment, Fujian Agriculture and Forestry University, Fuzhou 350002, China; 3 Key Laboratory of Biopesticide and Chemical Biology, Ministry of Education, Fuzhou 350002, China; 4 State Key Laboratory of Ecological Pest Control for Fujian and Taiwan Crop, Fuzhou 350002, China

**Keywords:** Eupelmids, key, new species, Chinese, egg parasitoids

## Abstract

*Anastatus* is the second-largest genus in the family Eupelmidae, with 150 species found worldwide and fourteen known species in China. Most species in this genus are the primary parasitoids of insect eggs, particularly those of Lepidoptera and Hemiptera, and several species have been used for the biological control of various insect pests. In this paper, seven new species of *Anastatus* Motschulsky, 1859 (Hymenoptera: Eupelmidae) from China are described, of which *A*. *caeruleus* Wang and Peng n. sp. and *A*. *garygibsoni* Zhou and Peng n. sp. were reared from the eggs of *Tessaratoma papillosa* (Hemiptera: Tessaratomidae), while *A*. *daiyunensis* Wang and Peng n. sp., *A*. *makrysourus* Zhou and Peng n. sp., *A*. *polikiarkoudus* Wang and Peng n. sp., *A*. *taibaiensis* Wang and Peng n. sp., and *A*. *zdenekbouceki* Zhou and Peng n. sp. were collected by malaise traps or sweeping. The new species are all described and illustrated based on female specimens, and the key to all 21 Chinese *Anastatus* species, based on females, is provided. Partial *COI* sequences of *A*. *caeruleus* n. sp., *A*. *garygibsoni* n. sp., and *A*. *taibaiensis* n. sp. are provided, and a phylogenetic reconstruction based on maximum likelihood analyses, while showing similar results at high taxonomic levels to our morphological feature classification results, provides additional information on the placement of *Anastatus* at the species level.

## Simple Summary

Correct taxonomic identification is essential for conducting successful biological research, especially of the important natural enemies of insects, such as *Anastatus,* a genus of parasitic wasps. In the present study, the key to all 21 Chinese *Anastatus* species, based on females, is provided, and seven new species are described and illustrated. We also used cytochrome oxidase I (*COI*) to identify and distinguish Chinese *Anastatus*. Seventeen sequences from seven *Anastatus* species were obtained and included in the GenBank database.

## 1. Introduction

Eight genera and sixty-six species of Eupelmidae from China have been recorded [1,2,3,4]. The genus *Anastatus* Motschulsky (Hymenoptera: Chalcidoidea: Eupelmidae) contains 150 species worldwide [1,2] and is classified into two subgenera: *A*. (*Anastatus*) and *A*. (*Cladanastatus*). Most species belong to the subgenus *A*. (*Anastatus*), which is characterized by a forewing that is almost always infuscate and patterned with a hyaline cross band or spots behind the marginal vein [5]. As members of the Eupelminae, females and males are strongly dimorphic in structure, with males largely retaining the plesiomorphic features of the mesosoma and females having highly modified mesosomal features that have evolved to enhance their jumping ability [6].

Most *Anastatus* species are the primary parasitoids of the eggs of other insects, and some have been reared from Coleoptera larvae and Diptera puparia [5,7]. Several species of *Anastatus* have been used for the biological control of various insect pests in China and elsewhere. For example, the native egg parasitoid *A. bifasciatus* (Geoffroy) is used for the biological control of *Halyomorpha halys* (Stål) (Hemiptera: Pentatomidae) in Europe [8,9]; the egg parasitoid *A. orientalis* Yang and Choi, native to China, is used for the biological control of *Lycorma delicatula* (Hemiptera: Fulgoridae) in the northeast U.S. [10,11]; and the solitary egg endoparasitoid *A. japonicus* Ashmead is used for the biological control of *Tessaratoma papillosa* (Drury) (Hemiptera: Pentatomidae) in China [12,13,14,15,16]. There is also research that indicates a high potential for the biological control of *Caligula japonica* Moore (Lepidoptera, Saturniidae) using *A*. *gansuensis* Chen and Zang [17]. Other strains of *Anastatus* have also been used as biological control [18]. The Chinese *Anastatus* species were recently studied and revised by Peng et al. Their research revealed that one species extensively used in China for the biocontrol of *Tessaratoma papillosa* was initially identified as *A. japonicus* Ashmead. However, their study indicates that, at least in southern China, this mostly results from the misidentification of *A. fulloi* Sheng and Wang. Furthermore, the correct interpretation of *A. japonicus* itself remains questionable, and many specimens in collections still need to be re-examined [2].

In this paper, seven new species from China were reported based on female specimens; six of them were collected from the Oriental Region and one species, *Anastatus taibaiensis* n. sp., was reported from the Palaearctic Region. All seven new species are described and illustrated, and the key to all twenty-one Chinese *Anastatus* species, based on females, is provided. Additionally, we provided seventeen sequences of cytochrome oxidase I (*COI*) in the mitochondrial genes of seven species of *Anastatus* from China, and an analysis based on the partial *COI* sequences of thirteen Chinese species was conducted. 

## 2. Materials and Methods

### 2.1. Sampling of the Specimens

The thirty-two specimens on which this study is based were obtained from two collections: the Biological Control Research Institute, Fujian Agriculture and Forestry University, Fuzhou, Fujian, China (FAFU), and the Hymenoptera Section, Natural History Museum, London, UK (NHMUK). The twenty-three specimens from FAFU were collected by malaise traps or sweeping from 2014 to 2017 and were then stored in 95% ethanol at −20 °C until DNA extraction. The nine specimens from the NHMUK were collected in 1983 and then mounted on paper points. 

### 2.2. Imaging

The descriptions were based on specimens that were examined with a Leica M165C stereo microscope (Leica Microsystems AG, Heerbrugg, Switzerland) and a Leica LED 5000 HDI dome light source (Leica Microsystems AG, Heerbrugg, Switzerland) and imaged with a Leica MC170 HD digital camera (Leica Microsystems AG, Heerbrugg, Switzerland) attached to the microscope. The serial images obtained were combined with Zerene Stacker 1.04 software (Richland, WA, USA). Adobe Photoshop CC2019 (Los Angeles, CA, USA) was used to edit the pictures and enhance their clarity.

### 2.3. Morphological Terminology

The terms used for their structure, sculpture, and color description follow Gibson [19]. The abbreviations used for structures in this paper are LOL = minimal distance between the anterior and posterior ocellus; MPOD = maximum diameter of the posterior ocellus; OOL = minimal distance between the posterior ocellus and the inner orbit; POL = minimal distance between the posterior ocelli; cc = costal cell of the forewing; mv = marginal vein of the forewing; pmv = postmarginal vein of the forewing; smv = submarginal vein of the forewing; and stv = stigmal vein of the forewing. The description of the new species was based on a holotype, and any major intraspecific variation based on paratypes is discussed under variation.

### 2.4. DNA Extraction, PCR Amplification, Sequencing, and Alignment

DNA extraction was carried out using a Micro Cell/Tissue DNA Kit (Biomarker Technologies, Beijing, China), following the manufacturer’s instructions, with some modifications: (i) the specimen was not crushed, but the gaster was pierced with an insect pin to create a hole, preserving the specimen while maximizing the quantity of DNA recovered; (ii) the incubation was prolonged to at least 12 h at 65 °C in a thermo-shaker; and (iii) the adsorption column was equilibrated at room temperature for 3 min before the eluent was added. The *COI* barcode fragment was amplified using the primers LCO1490 (5′-GGTCAACAAATCATAAAGATATTG-3′) and HCO2198 (5′-TAAACTTCAGGGTGACCAAAAAAT-3′) [20]. *COI* PCRs were performed in a 50 μL reaction volume containing 25 μL of 2× Gflex PCR Buffer (including Mg^2+^ and dNTP plus), 1 μL of Tks Gflex DNA Polymerase (Takara Biomedical Technology, Beijing, China), 0.5 μL of each primer (10 μM), 17 μL of ddH_2_O, and 6 μL of DNA template. The PCR conditions were as follows: initial denaturation at 94 °C for 5 min, followed by 35 cycles of 94 °C for 30 s, 49 °C for 30 s, and 72 °C for 1 min, with a final extension at 72 °C for 10 min. After electrophoresis on a 2% agarose gel, the *COI* PCR products were sent to Sangon Biotech (Shanghai, China) for bidirectional sequencing. Geneious R11 (Auckland, New Zealand) was used to check the quality of the peak pattern, manually correct and assemble the sequencing results, and export them in FASTA format. The sequences were aligned using ClustalW in Geneious R11 and checked to ensure they could be successfully translated into amino sequences without termination codons or frameshift mutations. All measured sequences were submitted to the NCBI, and additional sequences were downloaded from the NCBI, which had the accession numbers given in Table 1.

### 2.5. Species Delimitation Based on Molecular Data

To further validate our morphological identification, we used ASAP (Assemble Species by Automatic Partitioning) to analyze molecular data for species delimitation [21]. This analysis was conducted using the web server available at https://bioinfo.mnhn.fr/abi/public/asap/asapweb.html (accessed on 6 June 2024). In addition, genetic distances for each *COI* sequence were calculated using the Kimura 2-parameter (K2P) model in MEGA5.2 [22]. Pairwise similarity scores were determined with SDTv1.3 (Sequence Demarcation Tool Version 1.3) [23], which was also used to generate a color-coded matrix displaying these scores. 

### 2.6. Phylogenetic Analysis

In this study, a maximum likelihood (ML) tree was constructed using genus *Zaischnopsis* as an outgroup. The tree was based on seventeen newly obtained *COI* genes and eleven *Anastatus COI* genes downloaded from GenBank. The ML tree was inferred using IQ-TREE [24] in PhyloSuite [25] under the TIM + R2 + F model [26] with 5000 ultrafast bootstrap replicates [27] and the Shimodaira–Hasegawa-like approximate likelihood ratio test [28]. The ML tree was visualized using iTOL v.5 [29]. Nodal support for the ML analysis was assessed by the frequency of clade occurrence across the resampled datasets and is expressed as bootstrap values in %. BSs > 90% were considered to indicate significantly high nodal support, while BS values between 70 and 90% were regarded as moderately good support [30].

## 3. Results

### 3.1. COI Sequence Analysis

The *COI* sequence was successfully amplified from seventeen specimens across seven species: *Anastatus caeruleus* n. sp., *A*. *garygibsoni* n. sp., *A*. *taibaiensis* n. sp., *A*. *formosanus*, *A*. *dexingensis*, *A*. *gastropachae*, and *A*. *meilingensis*. Additionally, eleven *COI* sequences from six Chinese *Anastatus* species were downloaded from GenBank. The accession number and specific length of each sequence are listed in Table 1.

Following sequence alignment and the manual removal of upstream and downstream sequences, 624 base pair sequences were recovered for the studied species, representing a complete reading frame. This frame encodes 208 amino acids, and no termination codon was found. The sequences included 209 variable sites and 415 conserved sites. Among the variable sites, 169 were parsimony-informative, and 40 were singleton sites. 

**Figure 1:**
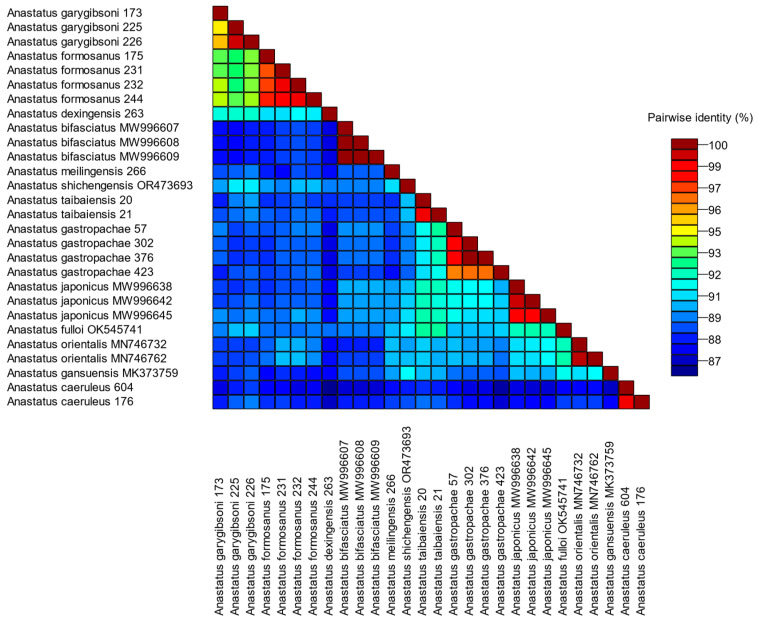
Color-coded matrix of pairwise similarity scores.

The genetic distance of thirteen *Anastatus* species was calculated using the *COI* gene with the Kimura-2 parameter model. The intraspecific genetic distance ranged from 0 to 5.5%, with the greatest intraspecific genetic distance of 5.5% observed in *A*. *garygibsoni*. The interspecific genetic distance ranged from 6.6% (between *A. garygibsoni* and *A*. *formosanus*) to 16.7% (between *A*. *caeruleus* and *A. dexingensis*) (Table S1). The sequence consistency relationships of *Anastatus* were visualized using SDTv1.3, as shown in Figure 1.

### 3.2. ASAP Species Definition Analysis

**Figure 2:**
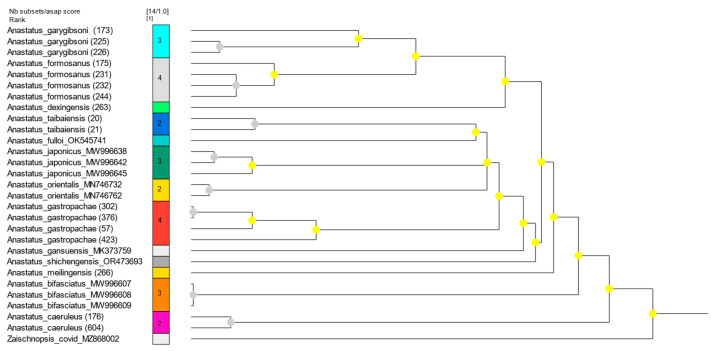
ASAP species definition analysis results, with nodes color-coded depending on their *p*-value (yellow: *p* > 0.1; gray: not applicable).

Based on the *COI* marker, the species boundaries were tested using ASAP analyses (Figure 2). The results from the ASAP species delimitation analysis indicated that the same species were well grouped, revealing a total of fourteen distinct species. The results align with our morphological identification.

### 3.3. Phylogeny of 13 Anastatus Species

**Figure 3:**
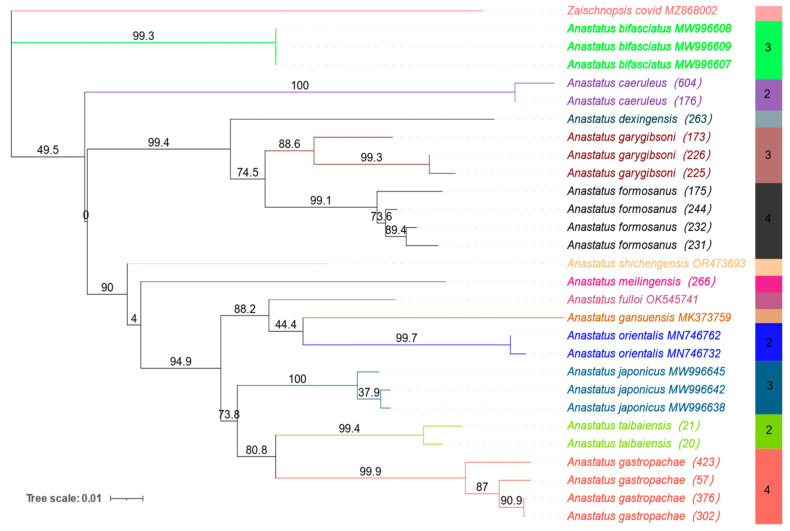
The ML tree (maximum likelihood) of *COI* sequences of *Anastatus*. Nodal support is assessed using bootstrap values.

As shown in Figure 3, *Zaischnopsis* is isolated as the outgroup, and all *Anastatus* species cluster independently in the ML tree, forming thirteen distinct branches without any irregularities, such as alternations or anomalies. *Anastatus garygibsoni* and *A*. *formosanus* are closely related and form a sister group, while *Anastatus taibaiensis* and *A. gastropachae* are closely related and positioned as sister species.

### 3.4. Description of New Species

#### 3.4.1. *Anastatus caeruleus* Wang and Peng n. sp. (Figure 4)

Zoobank:urn:lsid:zoobank.org:act:54A0A5C1-756A-4530-8FCA-5E5C94F13450

**Figure 4:**
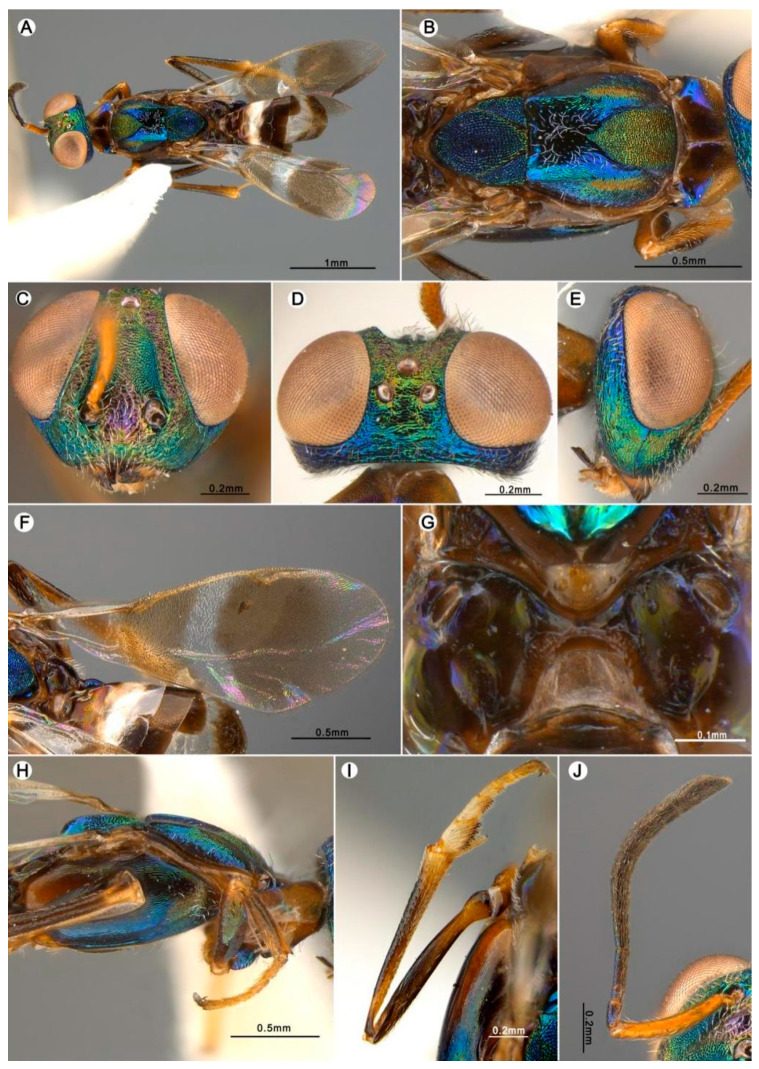
*Anastatus caeruleus* n. sp. (holotype): (**A**) body, dorsal; (**B**) mesosoma, dorsal; (**C**) head, frontal; (**D**) head, dorsal; (**E**) head, lateral; (**F**) forewing; (**G**) propodeum, dorsal; (**H**) mesosoma, lateral; (**I**) middle leg; (**J**) antenna.

**Type material.** Holotype ♀ (FAFU), Lichee Orchard, Longmen Road, Danzhou City, Hainan Province, China|19.5263° N, 109.5547° E, 140 m, 23 March 2017|Jiasheng Liu/DNA176. Paratypes: 1♀ same data as holotype, without DNA label; 1♀ (FAFU), the campus of Hainan University, Danzhou City, Hainan Province, China|19.5074° N, 109.4808° E, 153 m, 24 March 2017|Lingfei Peng/DNA604.

**Etymology.** The species name is derived from the Latin word ‘*caeruleus*’, which means blue, in reference to the blue metallic luster of the body. 

**Description.** HOLOTYPE, FEMALE, body length 3.3 mm. Head (Figure 4C–E) with extensive green and blue metallic luster at different angles; parascrobal region and interantennal region with purple to slightly golden metallic luster. Occiput, temple, vertex, and upper portion of parascrobal region bearing brown hair-like setae; lower face, lower portion of parascrobal region, and interantennal region with dense white hair-like setae (Figure 4C). Vertex, temple, and parascrobal region coriaceous to coriaceous-rugulose; lower face, gena, and vertex from posterior ocellus to occiput coriaceous and occiput reticulate rugose; scrobal depression coriaceous-rugulose. Mandibles and labial palps dark brown (Figure 4C). Antennae (Figure 4J) with scape yellowish brown; pedicel and flagellum dark brown. In frontal view (Figure 4C), head 1.3× as wide as high, distance between eyes below 2.5× distance between eyes above, scrobal depression ∩-like, arched dorsally, distance between scrobal depression and anterior ocellus 1.0× anterior ocellus diameter. In lateral view (Figure 4E), sub-spheroidal, malar space 0.4× eye height. In dorsal view (Figure 4D) width 2.3× length, with interocular distance about 0.3× head width; OOL:POL:LOL:MPOD = 2.5:9.5:7.0:7.0. Antenna (Figure 4J) relative length (and width): scape 110.4 (18.0); pedicel 23.2 (14.1); first to eighth funiculars: 12.0 (10.0), 36.0 (11.8), 35.0 (15.1), 30.0 (18.0), 25.0 (16.0), 24.0 (18.0), 23.2 (20.0), 20.1 (20.0); clava 70 (20.0).

Mesosoma (Figure 4A,B,H) dark brown with greenish blue or blue metallic luster at certain angles; pronotum dark brown dorsally, brown laterally; disc and around spiracles with bluish purple metallic luster, with few hair-like black setae around spiracles. Mesonotum (Figure 4B) dark, anterior part of medial lobe with yellowish green metallic luster, bearing tiny hair-like setae; posterior part with blue and purple metallic luster, bearing several white lanceolate setae; lateral lobe with yellowish green to greenish blue metallic luster; anterior part of medial lobe slightly convex, punctate reticulate; posterior part of medial lobe smooth, lateral lobe reticulate. Scutellar-axillar complex dark with greenish blue metallic luster, with four hair-like setae, scutellum drop-shaped with longitudinal reticulate sculpture, axillae triangular in dorsal view, anteromedial angles distinctly separated. Tegula brown, with several black hair-like setae. Prepectus (Figure 4H) light brown and bare. Acropleuron (Figure 4H) dark with greenish blue metallic luster, posterior third contrastingly paler (brown), setose with several white hair-like setae anteriorly, with shallow longitudinal reticulate-imbricate sculpture. Propodeum (Figure 4G) dark brown with slightly purple metallic luster. Macropterous, forewing (Figure 4F) disc covered with brown hair-like setae except one hyaline cross band behind marginal vein; costal cell bare dorsally, with a row of hair-like setae ventrally; basal cell sparsely setose with white hair-like setae; cc:mv:pmv:stv = 5.5:4.6:2.7:1.0. Legs (Figure 4A,H,I) dark brown except knees and tibiae, which are slightly yellowish brown; spur yellowish brown.

Gaster (Figure 4A) dark brown except for basal two segments, which are yellowish white, sparsely covered with dark brown hair-like setae. Ovipositor not visible in dorsal view.

**Variation.** The body length of the paratypes ranges from 3.3 to 3.4 mm. The scrobal depression is dorsally separated from the anterior ocellus by a distance of about 0.9–1.1× the longitudinal diameter of the ocellus. In frontal view, the head is 1.2–1.3× as wide as it is tall, and the distance between the eyes below is about 2.2–2.5× the distance between the eyes above. In lateral view, the malar space is 0.3–0.4× the eye height. In dorsal view, the head has a width of 1.9–2.3× the length, and the interocular distance is about 0.1–0.3× the head width. OOL:POL:LOL:MPOD = 2.1–2.5:6.0–7.4:6.0–7.4:5.8–7.0; cc:mv:pmv:stv = 5.5:4.6–5.3:2.7–2.9:1.0.

**Host.** Egg of *Tessaratoma papillosa* (Drury) (Hemiptera: Tessaratomidae).

**Distribution.** ORIENTAL REGION: China (Hainan).

**Diagnosis. ***Anastatus caeruleus* resembles *A. japonicus* but can be distinguished by the following characteristics: (1) The mesonotum of *A. caeruleus* is bright blue (Figure 4B), whereas it is generally green or golden green in *A. japonicus*; sometimes the posterior part of the medial lobe in *A. japonicus* may have a slightly blue metallic luster, but the anterior part of the medial lobe and scutellar-axillar complex are never blue. (2) The posterior third (no more than half) of the acropleuron is brown or slightly yellowish brown in *A. caeruleus*, which is paler than the dark blue anterior two-thirds of acropleuron (Figure 4H), whereas in *A. japonicus* at least the posterior two-thirds are brown or yellowish brown, and sometimes the entire acropleuron is brown. (3) The scrobal depression of *A. caeruleus* is dorsally separated from the anterior ocellus by a distance about equal to 0.9–1.1× of the longitudinal diameter of the ocellus (Figure 4D), but in *A. japonicus*, this distance is typically 1.2–2.0×, with the usual distance being around 1.5×.

**Remarks. ***Anastatus caeruleus* n. sp. and *A. garygibsoni* n. sp. were collected from an abandoned lichee orchard, which was soon after developed into a new urban area. This orchard contained a large range of eupelmids, with a total of six species collected, including *A*. *fulloi*, *A*. *formosanus*, *A*. *dexingensis*, and *A*. *shichengensis*. 

#### 3.4.2. *Anastatus daiyunensis* Wang and Peng n. sp. (Figure 5)

Zoobank:urn:lsid:zoobank.org:act:E50D7B79-B016-4ADB-BE0A-1CEB7ECB410A

**Figure 5:**
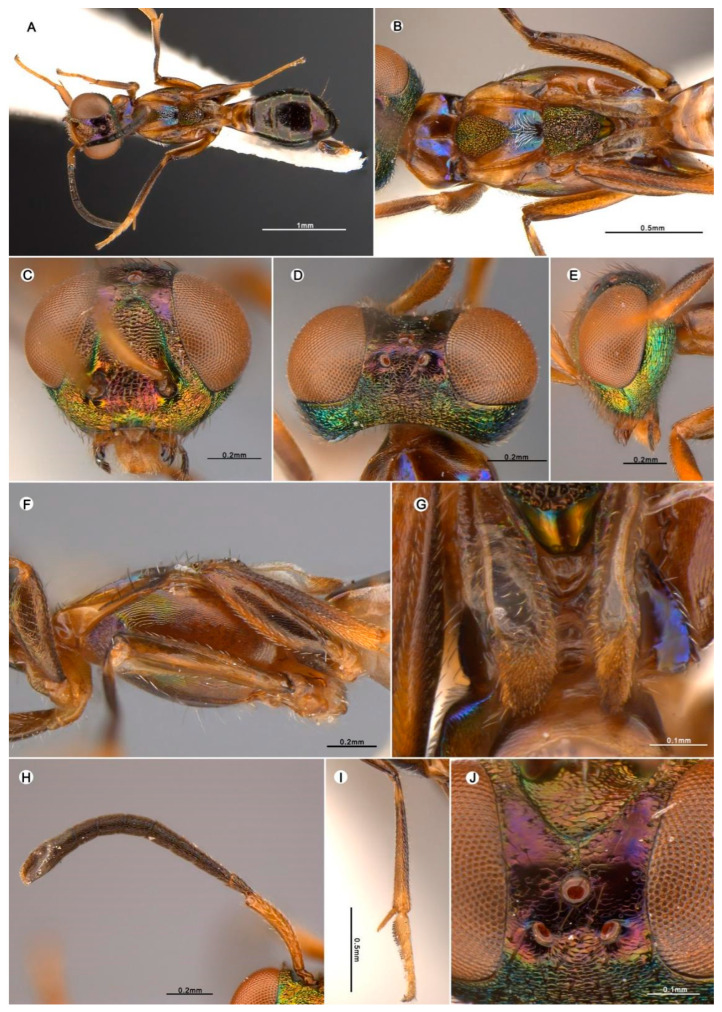
*Anastatus daiyunensis* n. sp. (holotype): (**A**) body, dorsal; (**B**) mesosoma, dorsal; (**C**) head, frontal; (**D**) head, dorsal; (E) head, lateral; (**F**) mesosoma, lateral; (**G**) propodeum; (**H**) antennae; (**I**) middle leg; (**J**) ocelli.

**Type material.** Holotype ♀ (FAFU), Houzhai, Daiyun Mountain Natural Reserve, Dehua County, Fujian Province, China|25.7121° N, 118.2518° E, 770 m, 15 July 2015|Malaise trap/DNA267. Paratypes 2♀ (FAFU), same data as holotype except the DNA numbers, DNA268 and DNA269. 

**Etymology.** Named after the mountain where the specimens are from. 

**Description.** HOLOTYPE, FEMALE, body length 3.2 mm. Head (Figure 5C–E) dark brown with green and purple metallic luster; vertex and frons with purple metallic luster; interantennal region with reddish purple metallic luster; lower face, gena, temple, and occiput with golden green metallic luster. Vertex and temple covered with brown hair-like setae, lower face with dense long lanceolate setae, interantennal region with lanceolate setae (Figure 5C). Vertex coriaceous (Figure 5D), frons smooth to slightly reticulate coriaceous (Figure 5J), upper face (Figure 5C) transitions to coarse reticulate rugose, lower face (Figure 5C) coriaceous rugose, the region from posterior ocellus to occiput and frons reticulate, scrobal depression transversely reticulate. Mandibles, maxillary, and labial palps black to dark brown (Figure 5C). Antennae (Figure 5H) with scape brown, pedicel and flagellum dark brown, pedicel has bluish green metallic luster. In frontal view (Figure 5C), head 1.4× as wide as high, distance between eyes below 2.2× distance between eyes above, scrobal depression ∩-like arched dorsally, distance between scrobal depression and anterior ocellus 1.05× anterior ocellus diameter. In lateral view (Figure 5E), sub-spheroidal, malar space 0.5× eye height. In dorsal view (Figure 5D), width 2.2× length, with interocular distance about 0.3× head width; OOL:POL:LOL:MPOD = 5.0:9.0:6.0:5.0. Antenna (Figure 5H) relative length (and width): scape 79.2 (12.1); pedicel 22.1 (12.1); first to eighth funiculars: 10.0 (10.0), 25.6 (13.6), 23.4 (15.0), 33.7 (17.0), 23.0 (16.0), 24.4 (16.2), 20.6 (17.0), 20.2 (18.6); clava 38.7 (22.7).

Mesosoma (Figure 5A,B,F) brown with blue and green metallic luster at some angles, pronotum dark brown with bluish purple metallic luster laterally; around spiracles black with black hair-like setae. Mesonotum (Figure 5B) yellowish brown, anterior part of medial lobe has golden green metallic luster, bearing white hair-like setae; posterior part with bluish green metallic luster, bearing white lanceolate setae; lateral lobe yellowish brown with several black lanceolate setae; anterior part of medial lobe slightly convex, punctate reticulate; posterior part of medial lobe and lateral lobe smooth. Scutellar-axillar complex brown with gold–green metallic luster, bare, scutellum drop-shaped with longitudinal reticulate sculpture, axillae triangular in dorsal view, anteromedial angles distinctly separated. Tegula (Figure 5F) light brown, with black lanceolate setae. Prepectus (Figure 5F) light brown and bare. Acropleuron (Figure 5F) yellowish brown with metallic luster, anterior third has dense white hair-like setae, posterior part bare, with shallow longitudinal reticulate-imbricate sculpture. Brachypterous, forewing (Figure 5G) hyaline except posterior third, light brown with brown lanceolate setae, without evident stigmal vein and postmarginal vein. Legs (Figure 5A,B,F) dark brown except knees and tibiae, which are slightly yellowish brown; mesotibiae and spur (Figure 5I) light brown.

Gaster (Figure 5A) dark brown except basal two segments which are yellowish brown, with sparse dark brown hair-like setae. Ovipositor not visible in dorsal view.

**Variation.** The body length is 2.4–3.3 mm. In frontal view, the head is 1.2–1.4× as wide as it is high, the distance between the eyes below is 1.6–2.2× the distance between the eyes above, the distance between scrobal depression and the anterior ocellus is 1.1–1.5× the anterior ocellus diameter. In lateral view, the malar space is 0.3–0.5× the eye height. In dorsal view, the width of the head is 1.7–2.2 × its length, and the interocular distance is about 0.1–0.3× the head width; OOL:POL:LOL:MPOD = 4.3–5.0:9.0–9.6:5.5–6.0:4.7–5.3. Relative length (and width) of antenna is scape 79.2–93.8 (12.1–12.5); pedicel 22.1–23.5 (12.1–12.5); and first to eighth funiculars: 10.0–11.3 (10.0), 25.6–25.8 (12–13.6), 23.4–24.5 (14.5–15.0), 27.3–33.7 (16.3–17.0), 23.0–24.8 (16.0–17), 24.4–26 (16.2–17.5), 20.6–22.5 (15.5–17.0), 20.2–20.5 (15.8–18.6); clava 38.7–53.5 (20.5–22.7).

**Host.** Unknown.

**Distribution.** ORIENTAL REGION: China (Fujian).

**Diagnosis. ***Anastatus daiyunensis* resembles *A. pariliquadrus* but can be distinguished by the following characteristics: (1) The upper portion of the parascrobal region and the area around the anterior ocellus are weakly coriaceous to smooth (Figure 5J). In contrast, in *A*. *pariliquadrus* and other brachypterous *Anastatus* species we examined, such as *A. flavaeratus*, *A. meilingensis*, and *A. zdenekbouceki* n. sp., this region is strongly coriaceous, granular, or reticulate. (2) The forewings of these two species are all blade-like and slightly curved, but in *A. daiyunensis* the apical part has denser, dark setae (Figure 5G), whereas *A. pariliquadrus* has sparser, light-colored setae. (3) The head shape of *A. daiyunensis* is lenticular from a lateral view, like most other *Anastatus* species, with a height about 1.4× that of its length, whereas in *A. pariliquadrus* the head is more square-shaped, with a height about 1.1 × its length.

#### 3.4.3. *Anastatus garygibsoni* Zhou and Peng n. sp. (Figure 6)

Zoobank:urn:lsid:zoobank.org:act:9B9D2BFC-1131-4082-9276-430808B81CF5

**Figure 6:**
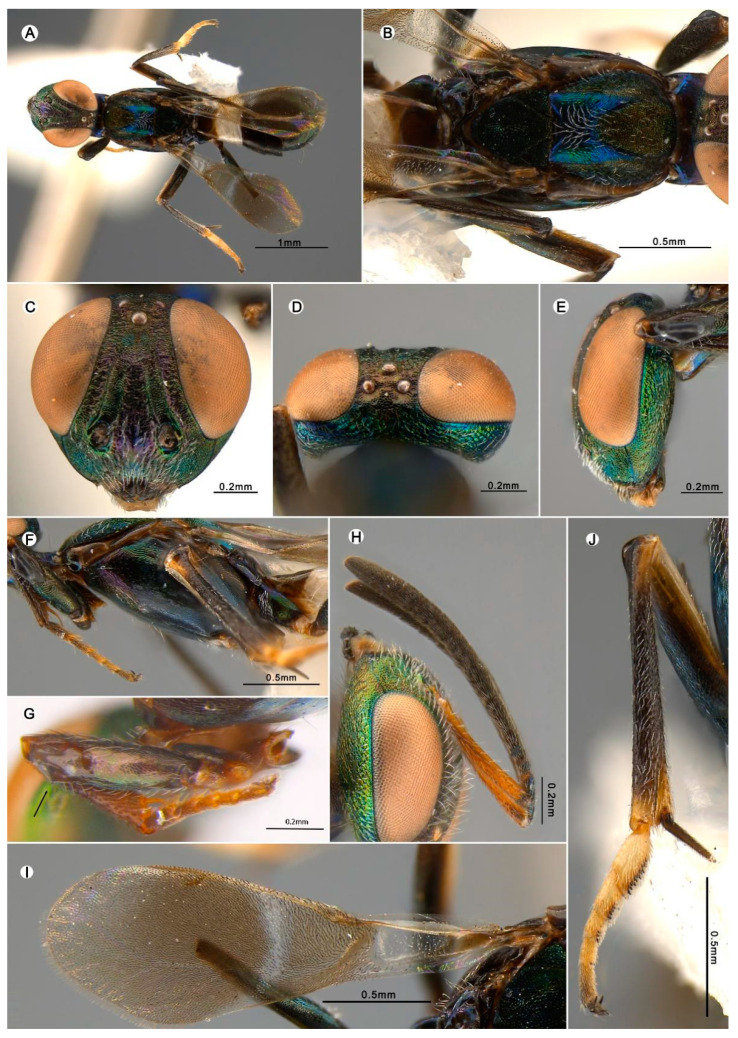
*Anastatus garygibsoni* n. sp. (holotype, except (**H**), which is from paratype): (**A**) body, dorsal; (**B**) mesosoma, dorsal; (**C**) head, frontal; (**D**) head, dorsal; (**E**) head, lateral; (**F**) mesosoma, lateral; (**G**) profemur (showing small spine); (**H**) antennae; (**I**) forewing; (**J**) middle leg.

**Type material.** Holotype ♀ (FAFU), lichee orchard, Longmen Road, Danzhou City, Hainan Province, China|19.5263° N, 109.5547° E, 140 m, 23 March 2017|Jiasheng Liu/DNA173. Paratypes 4♀ (FAFU), same data as holotype except the DNA numbers: DNA225, DNA226, DNA227, DNA281.

**Etymology.** The name is a tribute to Dr. Gary A.P. Gibson, in recognition of his great contribution to the world of Eupelmidae taxonomy. 

**Description.** HOLOTYPE, FEMALE, body length 4 mm. Head (Figure 6C–E) dark with extensive green metallic luster; vertex with greenish blue and purple metallic luster; frons with purple and dark green metallic luster; lower face, gena, and temple with bluish green metallic luster; occiput with greenish blue metallic luster; interantennal region with dark purple metallic luster. Vertex has brown hair-like setae, lower face has white lanceolate to hair-like setae, interantennal region has white lanceolate setae, temple has few white hair-like setae (Figure 6C). Vertex to frons from coriaceous to reticulate rugose, lower face and scrobal depression reticulate, posterior ocellus to occiput, gena and occiput slightly reticulate rugose. Mandibles, maxillary, and labial palps dark brown (Figure 6C). Antennae (Figure 6H) with scape yellowish brown, pedicel and flagellum dark brown; pedicel has slightly bluish purple metallic luster. In frontal view (Figure 6C), head 1.2× as wide as high, distance between eyes below 2.7× distance between eyes above, scrobal depression ∩-like arched dorsally, distance between scrobal depression and anterior ocellus 1.3× anterior ocellus diameter. In lateral view (Figure 6E), slightly lenticular, malar space 0.4× eye height. In dorsal view (Figure 6D), width 2.57× length, interocular distance about 0.38× head width; OOL:POL:LOL:MPOD = 2.5:10.0:7.5:6.5. Antenna (Figure 6H) relative length (and width): scape 100.6 (11.2); pedicel 21.6 (11.0); first to eighth funiculars: 11.4 (10.0), 23.7 (10.7), 28.5 (11.2), 26.8 (13.6), 26.2 (16.2), 20.7 (17.0), 19.7 (19.2), 16.7 (19.1); clava 49.5 (20.9).

Mesosoma (Figure 6A,B,F) black with bluish green or yellowish green metallic luster at some angles; pronotum bare, black to dark brown with purple metallic luster; disc and around spiracles have blue metallic luster. Mesonotum (Figure 6B) black, anterior part of medial lobe has brown metallic luster and bears several white hair-like setae; posterior part of medial lobe has greenish blue metallic luster and bears dense white hair-like setae; lateral lobe has green metallic luster, sparsely bearing white hair-like setae; anterior part of medial lobe slightly convex, punctate; lateral lobe reticulate coriaceous. Scutellar-axillar complex (Figure 6B) black with dark green metallic luster, bare, scutellum reticulate laterally with longitudinal punctate reticulate sculpture, axillae triangular in dorsal view, with anteromedial angles distinctly separated. Tegula black to dark brown with several black hair-like setae. Prepectus (Figure 6F) dark brown and bare. Acropleuron (Figure 6F) black with purple and green metallic luster, anterior third has dense white lanceolate setae, posterior part bare, with shallow longitudinal reticulate-imbricate sculpture. Macropterous, forewing (Figure 6I) disc covered with brown hair-like setae except one narrow hyaline cross band behind marginal vein, costal cell bare dorsally, with a row of hair-like setae ventrally, basal cell dorsally with several white hair-like setae; cc:mv:pmv:stv = 11.5:9.2:3.8:1.0. Legs (Figure 6A,F,J) black to dark brown except knees and mesotibiae, which are slightly yellowish brown, spur dark brown.

Gaster (Figure 6A) dark brown, except two basal segments which are yellowish white, and sparsely covered with dark brown hair-like setae. Ovipositor not visible in dorsal view.

**Variation.** The body length is 4–4.2 mm. In frontal view, the head is 1.1–1.2× as wide as it is high, and the distance between the eyes below is 2.3–2.9× the distance between the eyes above. In lateral view, the malar space is 0.24–0.4× the eye height. In dorsal view, the width of head is 2.1–2.57× its length, and the interocular distance is about 0.09–0.38× the head width. OOL:POL:LOL:MPOD = 2.5–3.3:8.4–10.0:6.9–7.5:5.8–6.5; cc:mv:pmv:stv = 10.5–12.5:7.5–9.5:3.5–4.1:1.0.

**Host.** Egg of *Tessaratoma papillosa* (Drury) (Hemiptera: Tessaratomidae).

**Distribution.** ORIENTAL REGION: China (Hainan).

**Diagnosis. ***Anastatus garygibsoni* and *A. dexingensis* both have a sharp tooth on their profemur, but can be distinguished by the following characteristics: (1) the forewing of *A. garygibsoni* has a narrow hyaline cross band, with the infuscate region behind the parastigma almost 10× the width of the hyaline band, and occasionally containing some dark setae within the hyaline band (Figure 6I). In contrast, in *A. dexingensis*, the infuscate region behind the parastigma is about 3–5× wider than the hyaline band. (2) The body length of *A. garygibsoni* is 4–4.2 mm, significantly longer than that of *A. dexingensis*, which is about 2.5 mm.

#### 3.4.4. *Anastatus makrysourus* Zhou and Peng n. sp. (Figure 7)

Zoobank:urn:lsid:zoobank.org:act:F61CCDB7-D25C-48B4-9F31-4081812BF68A

**Figure 7:**
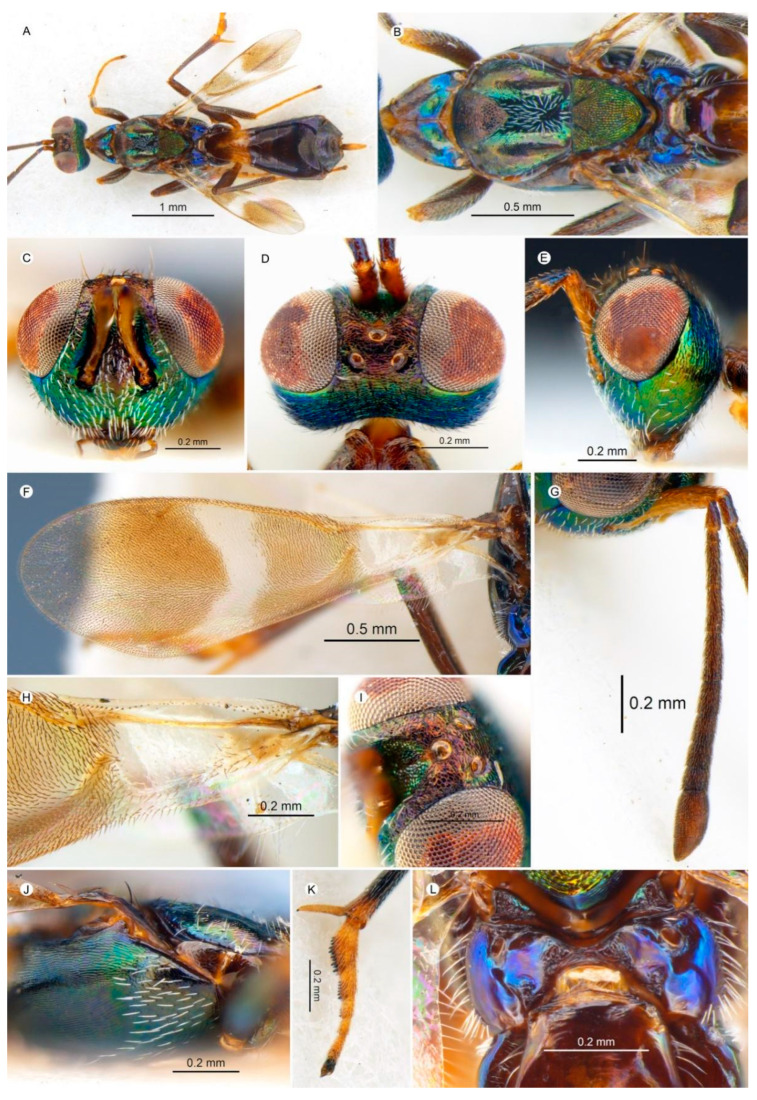
*Anastatus makrysourus* n. sp. (holotype, except (**F**,**H**), which are from the paratype NHMUK010353655): (**A**) body, dorsal; (**B**) mesosoma, dorsal; (**C**) head, frontal; (**D**) head, dorsal; (**E**) head, lateral; (**F**) forewing; (**G**) antennae; (**H**) costal cell and basal cell; (**I**) ocelli; (**J**) mesosoma, lateral; (**K**) middle leg; (**L**) propodeum.

**Type material.** Holotype ♀ (NHMUK), SE Hainan|Xing Long|23 May 1983/China: Hainan Isl.| May 1983|Bouček/NHMUK010353692. Paratypes 2♀ (NHMUK), same data as holotype except the collection numbers: NHMUK010353691, NHMUK010353655.

**Etymology.** From the Greek words *makrys* (long) and *oura* (tail), in reference to the ovipositor of the female being much longer than that of other new species. 

**Description.** HOLOTYPE, FEMALE, body length 3.6 mm. Head (Figure 7C–E) dark with extensive green metallic luster, while vertex, upper portion of parascrobal region, and interantennal prominence with more or less purple metallic luster. Vertex, temple and occiput covered with dark hair-like setae; gene with white hair-like to slightly lanceolate setae’ lower portion of parascrobal region, interantennal prominence, and lower face with distinct white lanceolate setae (Figure 7C). Vertex coriaceous and more transversely imbricate posteriorly; temple transversely imbricate; frons coriaceous; parascrobal region, interantennal region, and lower face coriaceous-rugose; gena slightly reticulate-imbricate. Mandibles, maxillary, and labial palps dark brown (Figure 7C). Antennae (Figure 7G) with scape yellowish brown, pedicel and flagellum dark brown. In frontal view (Figure 7C), head 1.32× as wide as high, distance between eyes below 2.29× distance between eyes above; scrobal depression dorsally M-like, arched (Figure 7I), and extended to anterior ocellus. In lateral view (Figure 7E), subspheroidal, malar space 0.46× eye height. In dorsal view (Figure 7D), width 1.75× length, with interocular distance about 0.33× head width; OOL:POL:LOL:MPOD = 1.0:2.2:1.5:1.5. Antenna (Figure 7G) relative length (and width): scape 86.6 (16.6); pedicel 26.5 (13.3); first to eighth funiculars: 10.0 (10.0), 37.3 (12.0), 37.4 (12.1), 40.0 (16.7), 26.7 (16.7), 24.0 (17.3), 22.7 (18.7), 20.0 (20.0); clava 45.5 (15.5). 

Mesosoma (Figure 7A,B,J) dark to black, with green-to-blue luster at some angles; pronotum dark brown with slightly green metallic luster laterally; disc and around spiracles have blue metallic luster, with three long hair-like setae and several short hair-like setae on disc. Mesonotum black, with anterior part of medial lobe having purple metallic luster and bearing several tiny hair-like setae; posterior part having long white hair-like setae; one row of long white hair-like setae along lateral lobe longitudinally; anterior part of medial lobe slightly convex; coriaceous to slightly reticulate posteriorly; posterior part of medial lobe smooth; lateral lobe evenly humped posteriorly and slightly coriaceous. Scutellar-axillar complex dark with green to slightly purple metallic luster, bare, scutellum with longitudinal reticulate sculpture, axillae triangular in dorsal view, with anteromedial angles distinctly separated. Tegula dark brown, with several black hair-like setae. Prepectus (Figure 7J) dark brown, bare. Acropleuron (Figure 7J) black with green metallic luster, setose with white hair-like setae anteriorly, with shallow longitudinal reticulate-imbricate sculpture. Propodeum (Figure 7L) black with bright purple metallic luster. Macropterous, forewing (Figure 7F,H) with costal cell dorsally bare except densely setose with dark setae in front of parastigma, with a row of brown hair-like setae ventrally; basal cell bare except dorsally, with a patch of brown hair-like setae basally and along mediocubital fold; disc with a hyaline cross band; cc:mv:pmv:stv = 10.5:8.6:2.9:1.0. Legs (Figure 7A,K) dark brown except knees and tibiae, which are slightly yellowish brown, spur yellowish brown. 

Gaster (Figure 7A) black except basal two segments that are yellowish brown, sparsely covered with dark brown hair-like setae. Ovipositor (Figure 7A) visible in dorsal view, 0.87× length of first metatarsomere. 

**Variation.** The body lengths of the two paratypes are 3.8 and 4.2 mm. From a dorsal view, the head widths are 1.57× and 1.60× the head length, and the interocular distances are about 0.29–0.33× the head widths; from a frontal view, the heads are about 1. 28× as wide as they are high, with the distance between the eyes below about 2.1× the distance between the eyes above. The other ratios of the head show no significant differences. The fore wing has cc:mv:pmv:stv = 6.9–7.5:7.5–7.8:2.5–2.9:1.0.

**Host.** Unknown.

**Distribution.** ORIENTAL REGION: China (Hainan).

**Diagnosis. ***Anastatus makrysourus* resembles *A. bifasciatus* but can be distinguished by the following characteristics: (1) The ovipositor of *A. makrysourus* is visible from a dorsal view and usually as long as the first segment of the metatarsus (Figure 7A), but in *A. bifasciatus*, the ovipositor is invisible. (2) In *A. makrysourus*, the dorsal margin of the scrobal depression extends to the anterior ocellus (Figure 7I). In contrast, in *A. bifasciatus*, the dorsal margin of the scrobal depression is about 2× the diameter of the anterior ocellus. (3) The basal region of the forewing and the area along the mediocubital fold are evenly setose in *A. makrysourus* (Figure 7H), whereas in *A. bifasciatus* these regions are bare. (4) The posterior part of the medial lobe in *A. makrysourus* is densely setose with white setae and arranged in a somewhat radiating pattern (Figure 7B), but in *A. bifasciatus* the posterior part of the medial lobe is sparsely setose with a few setae, and all are directed posteriorly. 

##### 3.4.5. *Anastatus polikiarkoudus* Wang and Peng n. sp. (Figure 8)

Zoobank:urn:lsid:zoobank.org:act:A9F2195D-0340-4338-A4F6-63ED50A69E85

**Figure 8:**
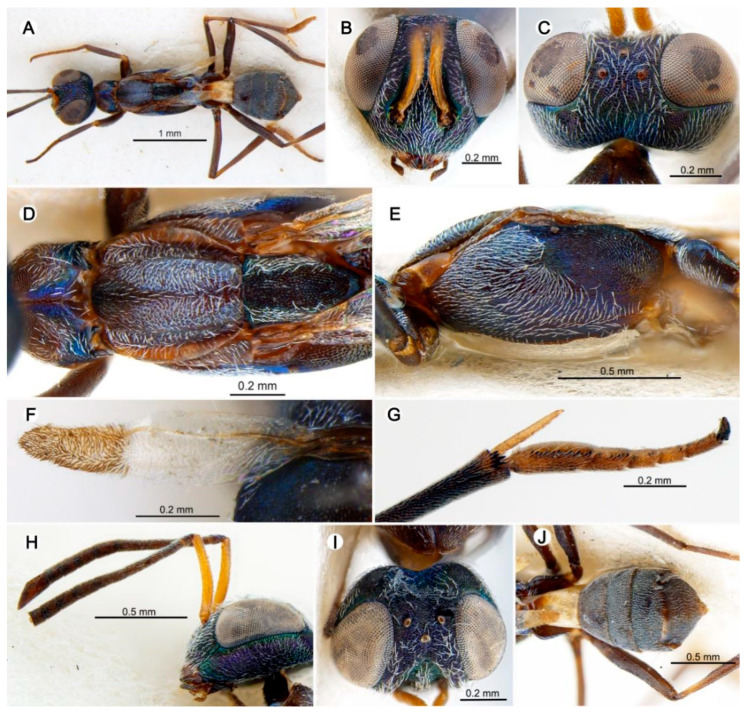
*Anastatus polikiarkoudus* n. sp. (holotype, except (**I**)): (**A**) body, dorsal; (**B**) head, frontal; (**C**) head, dorsal; (**D**) mesosoma, dorsal; (**E**) mesosoma, lateral; (**F**) forewing; (**G**) middle leg; (**H**) antennae; (**I**) ocelli; (**J**) gaster.

**Type material.** Holotype ♀ (NHMUK), Tien Fong Mts. Hainan Island, China|19 May 1983|Bouček/NHMUK010353700. Paratypes 2♀ (NHMUK), same data as holotype except the collection numbers: NHMUK010353629, NHMUK010353709.

**Etymology.** A combination of the Greek words *poliki arkouda* (polar bear), in reference to the appearance of the dense white hair setae of the body. 

**Description.** HOLOTYPE, FEMALE, body length 3.3 mm. Head (Figure 8B,C,I) dark in color with purple metallic luster; scrobal depression, margin of torulus, posterior orbit, and frontal malar suture with green metallic luster (Figure 8B). Head has dense lanceolate white setae except two bare patches behind compound eyes (Figure 8C). Vertex, face, and temple reticulate rugose, gene and occiput slightly longitudinal reticulate. Mandibles, maxillary, and labial palps dark brown (Figure 8B). Antennae (Figure 8H) with scape yellowish brown; pedicel and flagellum dark brown. In frontal view (Figure 8B), head 1.14× as wide as high, distance between eyes below 1.84× distance between eyes above, scrobal depression evenly ∧-like arched dorsally, and distance between scrobal depression and anterior ocellus 1.3× anterior ocellus diameter. In lateral view (Figure 8H), subspheroidal; malar space 0.36× eye height. In dorsal view (Figure 8C), width 1.8× length, with interocular distance about 0.17× head width; OOL:POL:LOL:MPOD = 1.0:3.0:2.2:1.0. Antenna (Figure 8H) relative length (and width): scape 85.5 (11.0); pedicel 23.3 (12.0); first to eighth funiculars: 8.0 (10.0), 27.5 (10.0), 28.5 (10.5), 30.1 (12.5), 24.1 (14.1), 20.0 (15.0), 17.0 (15.0), 15.0 (15.0); clava 44.5 (17.5).

Mesosoma (Figure 8D,E) dark in color with slight blue to purple luster, pronotum dark brown with dark blue metallic luster around spiracles, with lanceolate white setae on disc. Mesonotum dark brown with slight blue luster, with dense lanceolate white setae; anterior convex part of medial lobe punctate sculptured; lateral lobe slightly coriaceous-reticulate. Scutellar-axillar complex dark blue with slight green luster; scutellum ovate, bare, with longitudinal reticulate sculpture; axillae longitudinal narrow in dorsal view, with anteromedial angles distinctly separated and with same setae as mesonotum. Tegula dark brown, setose with white hair-like to slightly lanceolate setae. Prepectus (Figure 8E) brown to dark brown, bare. Acropleuron (Figure 8E) dark blue with slightly coppery luster: anterior half covered with dense lanceolate white setae extended to lower portion of acropleuron; posterior half from level of fore-wing base bare and with reticulated sculpture. Propodeum dark with blue to purple luster. Brachypterous (Figure 8F), apical third of forewing covered with dark brown setae, basal 2/3 hyaline, venation light brown, without evident stv and pmv; costal cell bare dorsally, basal cell densely setose. Legs (Figure 8A,G) dark brown except knees and tibiae, which are slightly yellowish brown, spur light brown. 

Gaster (Figure 8A) dark brown with dense lanceolate white setae, except two basal segments that are milky white to yellowish brown, and bare except a triangular parch of white hair-like setae near posterior margin of second tergum. Ovipositor (Figure 8A) not visible in dorsal view. 

**Variation.** The body lengths of the two paratypes are 2.1 and 3.5 mm. In the frontal view, the head is 1.1–1.22× as wide as it is high, the distance between eyes below is 1.84–1.96× the distance between the eyes above, and the distance between the scrobal depression and the anterior ocellus is 1.1–1.3× the diameter of the anterior ocellus; OOL:POL:LOL:MPOD = 1.0–1.1:3.0:2–2.2:1.0.

**Host.** Unknown.

**Distribution.** ORIENTAL REGION: China (Hainan).

**Diagnosis.** This species is unique and easily identifiable among all Chinese *Anastatus* due to the dense white setae covering most of its body. However, some parts of the body remain bare, including the scrobal depression, two patches behind the eyes, the anterior margin of the pronotum, the prepectus, the scutellum, the posterior part of the acropleuron, and the inner flank of the femur. 

##### 3.4.6. *Anastatus taibaiensis* Wang and Peng n. sp. (Figure 9)

Zoobank:urn:lsid:zoobank.org:act:180DEF63-196E-406A-8BA2-5BF88E1A8E8F

**Figure 9:**
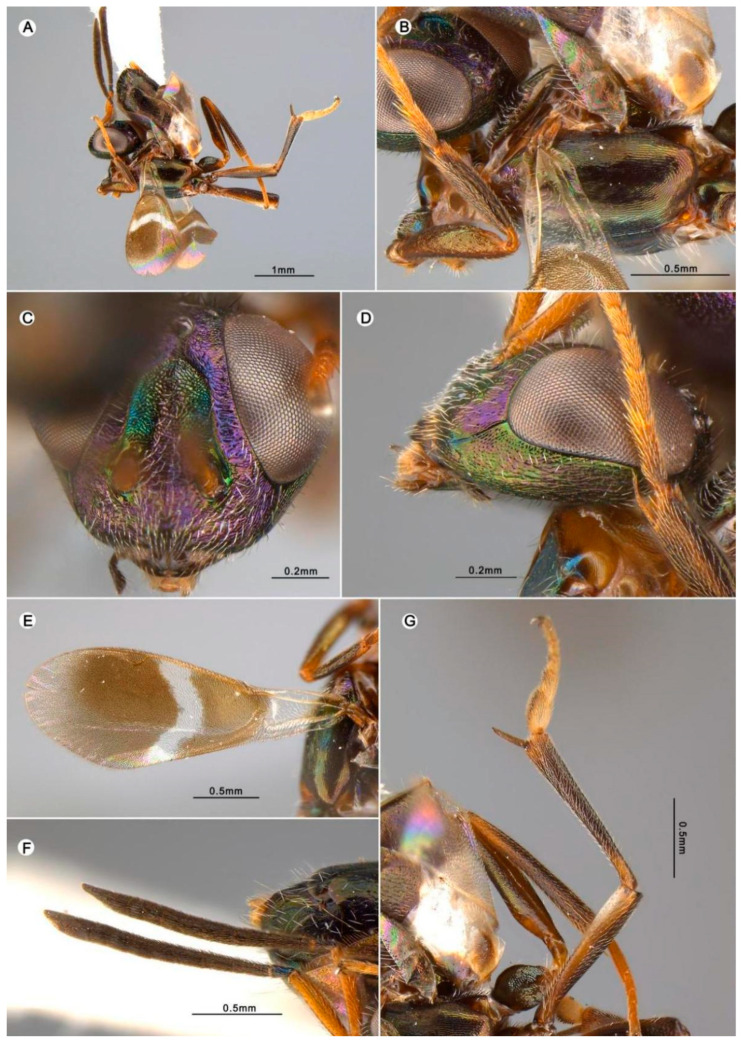
*Anastatus taibaiensis* n. sp. (holotype): (**A**) body, lateral; (**B**) mesosoma, lateral; (**C**) head, frontal; (**D**) head, lateral; (**E**) forewing; (**F**) antennae; (**G**) middle leg.

**Type material.** Holotype ♀ (FAFU), Haopingsi, Taibai Mountain, Baoji City, Shaanxi Province, China|Malaise trap, 22 August 2014|Yangzhao Fu/DNA21. Paratype 1♀ (FAFU), same data as holotype except the DNA number, DNA20. 

**Etymology.** From the mountain where the specimens are from.

**Description.** HOLOTYPE, FEMALE, body length 3.8 mm. Head (Figure 9C,D) dark with green and purple metallic luster; face and vertex with purple metallic luster; scrobal depression with bluish green metallic luster; gena, temple, and occiput with purplish green metallic luster. Vertex and temple covered with brown hair-like setae, parascrobal region with white hair-like setae, lower face with dense white hair-like setae (Figure 9C). Vertex to frons coriaceous sculptured to coarse transversely rugose, lower face reticulate, posterior ocellus to occiput, gena and temple slightly reticulate rugose, scrobal depression reticulate rugose. Mandibles black, maxillary palpus light brown, labial palps dark brown (Figure 9C). Antennae (Figure 9F) with scape yellowish brown, pedicel and flagellum dark brown; pedicel has slightly bluish purple metallic luster. In frontal view (Figure 9C), head 1.22× as wide as it is high, distance between eyes below 2.25× distance between eyes above, scrobal depression evenly ∩-like arched dorsally, distance between scrobal depression and anterior ocellus 1.0× anterior ocellus diameter. In lateral view (Figure 9D), subspheroidal, malar space 0.40× eye height. Antenna (Figure 9F) relative length (and width): scape 101.5 (17.5); pedicel 23.6 (12.5); first to eighth funiculars: 7.5 (10.0), 28.1 (11.2),27.7 (11.7), 28.2 (14.2), 27.9 (15.0), 23.6 (15.6), 22.3 (16.5), 21.0 (17.6); clava 31.0 (20.6).

Mesosoma (Figure 9B) dark brown with blue or greenish blue metallic luster. Mesonotum dark brown, with anterior part of medial lobe having brown metallic luster and posterior part having bluish purple metallic luster; lateral lobe light brown with purple metallic luster. Tegula light brown, bearing several black hair-like setae. Prepectus (Figure 9B) light brown and bare. Acropleuron (Figure 9B) dark brown with metallic luster: anterior part covered with white hair-like setae; posterior part bare, with shallow longitudinal reticulate-imbricate sculpture. Macropterous, forewing (Figure 9E) disc covered with brown hair-like setae except one hyaline cross band behind marginal vein; costal cell bare dorsally, with a row of brown hair-like setae ventrally; basal cell hyaline and covered with white hair-like setae dorsally; cc:mv:pmv:stv = 5.7:4.7:3.4:1.0. Legs (Figure 9A,B,G) dark brown except knees and tibiae, which are slightly yellowish brown, spur brown.

Gaster (Figure 9A) dark brown, except two basal segments that are white, and sparsely covered with dark brown hair-like setae. Ovipositor (Figure 9A) not visible in dorsal view.

**Variation.** The paratype was broken and unavailable for measurement. 

**Host.** Unknown.

**Distribution.** ORIENTAL REGION: China (Shaanxi).

**Diagnosis.** *Anastatus taibaiensis* resembles *A. gansuensis* but can be distinguished by the following characteristics: (1) In *A. taibaiensis*, the face is bright purple from a frontal view except for the scrobal depression, which is bluish green (Figure 9C), but in *A. gansuensis*, the face is mostly green with more or less purple or blue metallic lusters. (2) In *A. taibaiensis*, the mesotarsus is pale and the mesotibial apical spur is dark brown (Figure 9G). In contrast, in *A. gansuensis*, the mesotarsus is pale and the two basal segments are slightly darker than the two apical segments, and the mesotibial apical spur is as pale as the mesotarsus.

##### 3.4.7. *Anastatus zdenekbouceki* Zhou and Peng n. sp. (Figure 10)

Zoobank:urn:lsid:zoobank.org:act:2939F560-5095-44C3-9E98-C2FB970A68A4

**Figure 10:**
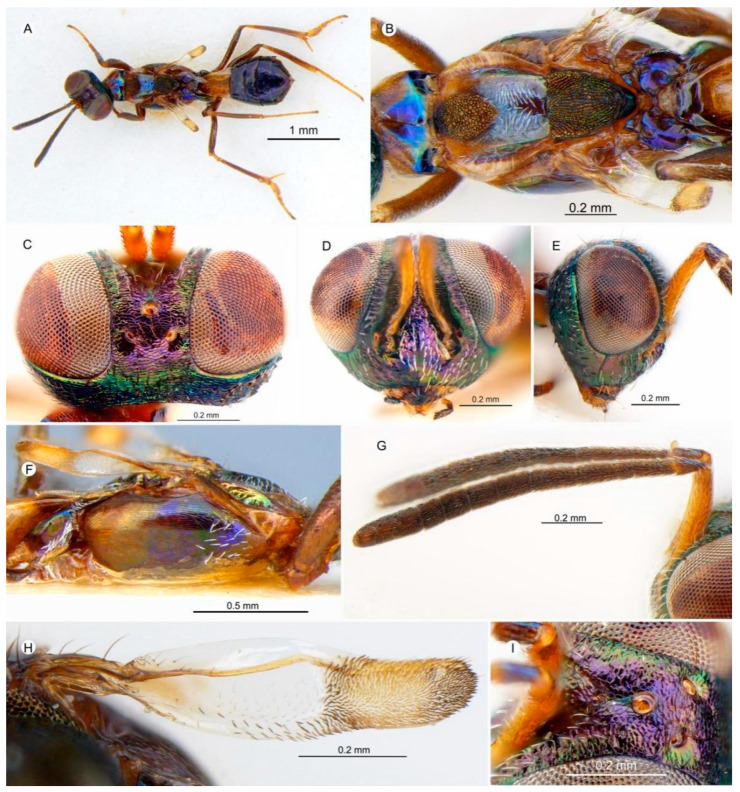
*Anastatus zdenekbouceki* n. sp. (holotype): **(A**) body, dorsal; (**B**) mesosoma, dorsal; (**C**) head, dorsal; (**D**) head, frontal; (**E**) head, lateral; (**F**) mesosoma, lateral; (**G**) antennae; (**H**) forewing; (**I**) ocelli and scrobal depression.

**Type material.** Holotype ♀ (NHMUK), SE Hainan|Xing Long|23 May 1983/China: Hainan Isl.| May 1983|Bouček/NHMUK010353644. Paratypes 2♀ (NHMUK), same data as holotype except the collection numbers: NHMUK010353640, NHMUK010353641.

**Etymology.** The name is a tribute to the Czech entomologist Dr. Zdeněk Bouček (1924–2011), in recognition of his great contribution to the taxonomy of Chalcidoidea. 

**Description.** HOLOTYPE, FEMALE, body length 3.0 mm. Head (Figure 10C–E) mostly dark with extensive green metallic luster, while vertex, parascrobal region, interantennal prominence, and lower face have strong purple metallic luster. Vertex, temple, and occiput covered with dark hair-like setae; gene and parascrobal region with white hair-like to lanceolate setae; interantennal prominence with distinct white lanceolate setae (Figure 10D). Vertex (Figure 10C) coriaceous and more transversely imbricate posteriorly; temple transversely imbricate; frons coriaceous (Figure 10I); parascrobal region, interantennal region, and lower face coriaceous-rugose; gena slightly reticulate-imbricate. Mandibles, maxillary, and labial palps dark brown (Figure 10D). Antennae (Figure 10G) with scape yellowish brown; pedicel and flagellum dark brown. In frontal view (Figure 10D), head 1.38× as wide as it is high, distance between eyes below 2.36× distance between eyes above, scrobal depression dorsally ∩-like arched, and distance between scrobal depression and anterior ocellus 1.1× anterior ocellus diameter (Figure 10I). In lateral view (Figure 10E), subspheroidal, malar space 0.27× eye height. In dorsal view (Figure 10C), width 1.76× length, with interocular distance about 0.30× head width; OOL:POL:LOL:MPOD = 1.0:2.3:1.7:1.0. Antenna (Figure 10G) with relative length (and width): scape 85.0 (12.5); pedicel 20.2 (11.0); first to eighth funiculars: 9.0 (10.0), 28.0 (10.0), 25.0 (13.5), 30.0 (14.5), 22.5 (14.1), 20.0 (16.5), 17.0 (17.0), 17.0 (18.0); clava 45.5 (15.5). 

Mesosoma (Figure 10A,B,F) brown with slight blue-to-purple luster at some angles; pronotum brown with green and blue metallic luster laterally; disc and around spiracles have purple metallic luster and are almost bare, with a few short hair-like setae on disc. Mesonotum (Figure 10B) with anterior third yellowish brown that gradually becomes dark brown posteriorly and blue and green metallic luster at some angles, anterior part of medial lobe bare, posterior part covered with long white hair-like setae, one row of long white hair-like setae along lateral lobe longitudinally, anterior part of medial lobe slightly convex, punctate sculptured, posterior part of medial lobe smooth, lateral lobe strongly ridged posteriorly and slightly coriaceous. Scutellar-axillar complex dark with green metallic luster, bare; scutellum drop-shaped with longitudinal reticulate sculpture, axillae longitudinal triangular in dorsal view, with anteromedial angles distinctly separated. Tegula light brown, with several black hair-like setae. Prepectus (Figure 10F) brown and bare. Anterior half of acropleuron (Figure 10F) dark purple and setose with few white hair-like setae; posterior half dark and yellowish brown, with shallow longitudinal reticulate-imbricate sculpture. Propodeum (Figure 10B) dark with bright bluish purple metallic luster. Brachypterous (Figure 10H): apical third of forewing covered with a patch of orange–brown setae surrounded with dark brown setae; mediocubital fold and cubital area with rows of brown setae, without evident stv and pmv; basal cell slightly brown; costal cell dorsally bare. Legs (Figure 10A) dark brown except knees and tibiae, which are slightly yellowish brown, spur light brown. 

Gaster (Figure 10A) dark brown, except two basal segments that are yellowish brown, and sparsely covered with dark brown hair-like setae. Ovipositor (Figure 10A) not visible in dorsal view. 

**Variation.** The body length is 3.0–3.3 mm. In the frontal view, the head is 1.36–1.38× as wide as it is high, and the distance between the eyes below is about 2.3–2.44× the distance between the eyes above. The distance between the dorsal margin of the scrobal depression and the anterior ocellus is about 1.0–1.1× the diameter of the anterior ocellus. In the dorsal view, the width of head is 1.6–1.8× its length, and the interocular distance is about 0.25–0.30× the head width. The OOL:POL:LOL:MPOD = 1.0–1.1:2.0–2.3:1.7:1.0. The antenna has the following relative lengths (and widths): scape 65.0–85.0 (10.2–12.5); pedicel 15.2–20.2 (10.4–11.0); the first to the eighth funiculars: 9.0–9.2 (10.0), 25.0–28.0 (10.0), 25.0–29.0 (13.5), 30.0–32.2 (14.5), 22.0–22.5 (14.1), 20.0–21.5 (16.5), 15.2–17.0 (17.0), 15.1–17.0 (18.0); clava 40.3–45.5 (13.5–15.0).

**Host.** Unknown.

**Distribution.** ORIENTAL REGION: China (Hainan).

**Diagnosis. ***Anastatus zdenekbouceki* resembles *A. meilingensis* but can be distinguished by the following characteristics: (1). In *A. zdenekbouceki*, the apex of the forewing is rounded (Figure 10H), but in *A. meilingensis* it is truncate. (2). The mesoscutum, scutellum, and axilla of *A. zdenekbouceki* display a slightly greenish golden brown metallic luster (Figure 10B), whereas in *A*. *meilingensis* these areas have a strong green coloration. (3). The spur of *A. zdenekbouceki* is light-colored, similar to the mesotarsus (Figure 10A), while in *A. meilingensis* the spur is dark brown, contrasting with the light-colored mesotarsus. 

### 3.5. Key to Anastatus Species from China Based on Female

1 Brachypterous, forewing variably reduced, extending no more than two-thirds the length of gaster (Figure 8F and Figure 10H)……………………………………………..………2
- Macropterous, forewing not reduced, extending at least to apex of gaster (Figure 4A and Figure 7A)…………………………………………………………………………………………8
2(1) Forewing discal region containing distinct hyaline cross band behind marginal vein…………………………………………………………………………………………3
- Forewing discal region containing no hyaline cross band behind marginal vein (Figure 8F and Figure 10H)…………………………………………………………………………………4
3(2) Forewing with distinct stigmal vein projecting into disc and comparatively long marginal vein, which is longer than half of the submarginal vein; mesoscutum, scutellum, and axilla lacking metallic luster……*Anastatus flavaeratus* Peng and Tang
- Forewing without evident stigmas vein or with short stigma vein that is no longer than width of marginal vein and appressed to post marginal vein; marginal vein shorter than half of submarginal vein; mesoscutum, scutellum, and axilla with strong green-to-blue metallic luster……………………………*Anastatus gastropachae* Ashmead
4(2) Body covered with dense white setae, except scrobal depression, two patches behind eye of head (Figure 8C), scutellum (Figure 8D), and posterior portion of acropleuron (Figure 8E) of mesosoma…………………………………….*Anastatus polikiarkoudus* n. sp.
- Body setation different from the species above…………………………………………..5
5(4) Upper portion of parascrobal region and region around anterior ocellus weakly coriaceous to smooth (Figure 5C,J) …………………………*Anastatus daiyunensis* n. sp.
- Upper portion of parascrobal region and region around anterior ocellus rugose or reticulate (Figure 8C,I)…………………………………………………………………………6
6(5) Forewing discal region covered with dense, light-colored setae………………………………………………*Anastatus pariliquadrus* Peng and Tang
- Forewing discal region covered with dense, dark brown setae except for region of orangish setae medially……………………………………………………………………7
7(6) Forewing with rounded apex (Figure 10H); mesoscutum, scutellum, and axilla with slightly greenish golden brown metallic luster (Figure 10B); spur light brown, same color as mesotarsus…………………………………………*Anastatus zdenekbouceki* n. sp.
- Forewing with apex somewhat truncate; mesoscutum, scutellum, and axilla with strong green metallic luster; spur dark brown, mesotarsus light brown……………………………………………….*Anastatus meilingensis* Sheng and Yu
8(1) Forewing containing two hyaline spots covered with white setae behind marginal vein separated by infuscated region with dark setae ……………………………………………………………*Anastatus echidna* (Motschulsky)
- Forewing with complete hyaline cross band behind marginal vein, wide or narrow, sometimes with some dark setae within hyaline region medially……………………9
9(8) Ventral profemur with one blunt to sharp tooth roughly within apical third (Figure 6G)….……………………………………………………………………………………….10
- Ventral profemur without tooth (Figure 7B and Figure 9B)……………………………………14
10(9) Mesoscutum with anterior convex part of medial lobe mesh-like coriaceous to pustulate over anterior half and mesh-like reticulate over posterior half; posterior concave part only sparsely setose …………………………*Anastatus colemani* Crawford
- Mesoscutum with anterior convex part of medial lobe entirely mesh-like reticulate; posterior concave part densely setose……………………………………………………11
11(10) Profemur with ventral margin abruptly angulate, forming a blunt tooth………………………………..……………*Anastatus shichengensis* Sheng and Wang
- Profemur with ventral tooth distinct and spine-like……………………………………12
12(11) Forewing with narrow hyaline cross band, infuscate region behind parastigma almost 10× wider than cross band, and many dark setae within hyaline region (Figure 6I); spur dark brown (Figure 6J)………………………………*Anastatus garygibsoni* n. sp.
- Forewing with wide hyaline cross band, infuscate region behind parastigma about 2× to 3× wider than cross band (Figure 4F, Figure 7F and Figure 9E); spur dark brown (Figure 6J and Figure 9G) or as pale as metatarsus (Figure 4I)……………………………………………13
13(12) Mesoscutum lateral lobe with differentiated bare band of more-or-less mesh-like coriaceous sculpture anterior to posteromedian carina; scrobal depression usually with distinct dorsal margin ……………………………..*Anastatus formosanus* Crawford
- Mesoscutum lateral lobe almost evenly setose and more-or-less uniformly roughened; reticulate-imbricate to reticulate-rugose anterior to posteromedian carina; indistinct scrobal depression usually with dorsal margin………………………………………………*Anastatus dexingensis* Sheng and Wang
14(9) Mesoscutum with anterior convex part of medial lobe at least extensively mesh-like coriaceous, at most distinctly reticulate only posteriorly…………………….………15
- Mesoscutum with anterior convex part of medial lobe mostly to entirely mesh-like reticulate……………………………………………………………………………………16
15(14) Scrobal depression dorsally ∩-like and arched and separated with anterior ocellus at least equal to 2× diameter of anterior ocellus; forewing basal region entirely bare; ovipositor not visible in dorsal view…………………….*Anastatus bifasciatus* (Geoffroy)
- Scrobal depression dorsally M-like and arched (Figure 7D) and extended to anterior ocellus; forewing basal region evenly setose along mediocubital fold and base of basal cell; ovipositor visible in dorsal view, elongated, as long as first segment of metatarsus……………………………………………………*Anastatus makrysourus* n. sp.
16(14) Acropleuron with posterior two-thirds to one-third paler (orange, brown, or yellow) than dark mesoscutum………………..…………………………………………………..17
- Acropleuron with posterior portion uniformly as dark as mesoscutum……………18
17(16) Acropleuron distinctly paler than dark mesoscutum over at least about posterior half; scrobal depression dorsally separated from anterior ocellus by distance equal to 1.2–2.0× longitudinal diameter of ocellus……..…………*Anastatus japonicus* Ashmead
- Acropleuron with only posterior third paler (brown) than dark mesoscutum (Figure 4H); scrobal depression dorsally separated from anterior ocellus by distance equal to 0.9–1.1× longitudinal diameter of ocellus (Figure 4D)…………*Anastatus caeruleus* n. sp.
18(16) Face bright purple in frontal view except scrobal depression which is bluish green (Figure 9C), mesotarsus pale, and mesotibial apical spur dark brown (Figure 9G) ………………………………………………………………*Anastatus taibaiensis* n. sp.
- Face mostly green with more-or-less purple or blue metallic luster; mesotarsus pale or two basal segments slightly darker than two apical segments; mesotibial apical spur as pale as mesotarsus…………………………………………………….…………19
19(18) Mesoscutum with posterior concave part setose medially for a width about equal to width of the bare region on either side; flagellum with all funiculars longer than they are wide……………………………………………*Anastatus gansuensis* Chen and Zang
- Mesoscutum with posterior concave part setose for almost entire width; flagellum with at least apical funicular quadrate to slightly transverse………………………20
20(19) Mesotarsus uniformly yellowish-to-white in contrast to dark pegs; procoxa with at least ventral surface usually similarly pale to lateral panel of pronotum……………………………………………*Anastatus orientalis* Yang and Choi
- Mesotarsus sometimes entirely infuscate, similar to dark pegs, but at least two basal tarsomeres variably conspicuously infuscate over at least dorsal and posterior surfaces; procoxa entirely dark, much darker than lateral surface of pronotum………………………………………………*Anastatus fulloi* Sheng and Wang

## 4. Discussions

In the taxonomic study of Eupelmidae, mitochondrial cytochrome oxidase I (*COI*) gene sequence divergence is commonly used for species identification [31]. Generally, the intraspecific genetic distance for *COI* sequences ranges from 0% to 2%. However, studies by Gibson et al. [32] show that, in some cases, geographical isolation leads to greater differences. For example, the genetic distance between the Japanese *Eupelmus kiefferi* and those from the mainland is slightly greater than 5%, which is larger than the maximum intraspecific distance of 4.8% found for *E. annulatus* by Al khatib et al. [33]. Similar results were observed in *Eupelmus* (*Macroneura*) studied by Fusu [34]: their intraspecific genetic distances range from 0.3% in *E. barai* to 5.2% in *E. muellneri*, while their interspecific genetic distances vary from 7.6% (between *E. atropurpureus* and *E. vladimiri*) to 15.3% (between *E. fulvipes* and *E. messene*). 

We obtained an interesting result in our study: there is significant genetic diversity within the single species *Anastatus garygibsoni*, with genetic distances of 5.5% observed, which is larger than the 5% observed in *E. kiefferi* and 5.2% in *E. muellneri*. This suggests substantial genetic variation within *A. garygibsoni*. The high genetic diversity within *Anastatus garygibsoni* may be due to factors such as geographical isolation, niche differentiation within its range, or prolonged independent evolution leading to considerable genetic differentiation among its populations. Meanwhile, the genetic distance between *A. garygibsoni* and *A. formosanus* is only slightly higher, at 6.6%, indicating close genetic relatedness and challenging traditional thresholds for species delimitation. This proximity raises questions about the genetic divergence processes in *Anastatus* species and might suggest the presence of cryptic speciation within *A. garygibsoni* or recent divergence between these species.

Our *COI* analysis results effectively supported specimen clustering; however, using empirical thresholds (e.g., 2% and 3%) for clustering may lead to an overestimation of species diversity. Ensuring a minimum interspecific genetic distance of 2% or more among congeneric species can help mitigate this overestimation [31]. It is important to note that species delimitation should not rely solely on genetic distance; morphological characters, ecological niches, and behavioral traits should also be considered in comprehensive assessments. The results from the ASAP analysis, along with the morphological identification, confirm that *A. garygibsoni* is a new species.

## 5. Conclusions

In this study, seven new species of *Anastatus* from China were described: *A*. *caeruleus*, *A*. *garygibsoni*, *A*. *daiyunensis*, *A*. *makrysourus*, *A*. *polikiarkoudus*, *A*. *taibaiensis,* and *A*. *zdenekbouceki.* All the new species are described and illustrated based on female specimens, and the key to all twenty-one known Chinese *Anastatus* species, based on females is, provided. We also provide DNA barcodes for the seven species collected in China, four of which were barcoded for the first time. We believe that molecular data are a valuable tool, providing new insights into the diagnosis of eupelmid species, especially when combined with reliable morphological characteristics. We found good agreement between the species definitions based on molecular and morphological data. However, the low genetic divergence and the small morphological differences between some eupelmid species also demonstrate the importance of using an integrative taxonomic approach [34,35]. Further ecological data and multilocus molecular data are needed for accurate species identification.

## Figures and Tables

**Table 1:** Accession numbers and lengths of *COI* sequences of each species.

Numbers	Species	Accession Numbers	Specimen ID	Length of Sequences (bp)
1	*Anastatus taibaiensis* n. sp.	PQ053069	20	624
2	*Anastatus taibaiensis* n. sp.	PQ053070	21	628
3	*Anastatus caeruleus* n. sp.	PQ165807	604	652
4	*Anastatus caeruleus* n. sp.	PQ053071	176	652
5	*Anastatus garygibsoni* n. sp.	PQ053072	173	652
6	*Anastatus garygibsoni* n. sp.	PQ053073	225	652
7	*Anastatus garygibsoni* n. sp.	PQ053074	226	652
8	*Anastatus formosanus* Crawford	PQ165808	175	652
9	*Anastatus formosanus* Crawford	PQ165809	231	652
10	*Anastatus formosanus* Crawford	PQ165810	232	652
11	*Anastatus formosanus* Crawford	PQ165811	244	652
12	*Anastatus dexingensis* Sheng and Wang	PQ165812	263	652
13	*Anastatus meilingensis* Sheng and Yu	PQ165813	266	652
14	*Anastatus gastropachae* Ashmead	PQ165814	57	652
15	*Anastatus gastropachae* Ashmead	PQ165815	302	652
16	*Anastatus gastropachae* Ashmead	PQ165816	376	652
17	*Anastatus gastropachae* Ashmead	PQ165817	423	638
18	*Anastatus shichengensis* Sheng and Wang	OR473693		652
19	*Anastatus gansuensis* Chen and Zang	MK373759		652
20	*Anastatus bifasciatus* (Geoffroy)	MW996607		652
21	*Anastatus bifasciatus* (Geoffroy)	MW996608		652
22	*Anastatus bifasciatus* (Geoffroy)	MW996609		652
23	*Anastatus fulloi* Sheng and Wang	OK545741		1542
24	*Anastatus japonicus* Ashmead	MW996638		652
25	*Anastatus japonicus* Ashmead	MW996642		652
26	*Anastatus japonicus* Ashmead	MW996645		652
27	*Anastatus orientalis* Yang and Choi	MN746732		652
28	*Anastatus orientalis* Yang and Choi	MN746762		652
29	*Zaischnopsis covid* Peng and Tang	MZ868002		759

## Data Availability

All DNA sequences are available in GenBank (*COI* accession numbers: PQ053069-PQ053074 and PQ165807-PQ165817).

## Supplementary Materials

The following supporting information can be downloaded at https://www.mdpi.com/article/10.3390/insects15110893/s1, Table S1. Pairwise genetic distances for *COI* gene sequences of *Anastatus* species.
