# Supplementary material for: Seven New Species of Anastatus Motschulsky (Hymenoptera: Chalcidoidea: Eupelmidae) from China Identified Based on Morphological and Molecular Data
Source: Insects. 2024 Nov 15;15(11):893. doi: 10.3390/insects15110893 (PMC11594841; doi:10.3390/insects15110893)
Supplement: Supplementary file 1 [file insects-15-00893-s001.zip › Table_S1.pdf]

Table S1. Pairwise genetic distances for *COI* gene sequences of *Anastatus*.1~2 = *A. taibaiensis* n. sp.; 3~4 = *A. caeruleus* n. sp.; 5~7 = *A. garygibsoni* n. sp.; 8~11 = *A. formosanus*.; 12 = *A. dexingensis*; 13 = *A. meilingensis*; 14~17 = *A. gastropachae*; 18 = *A. shichengensis*; 19 = *A. gansuensis*; 20~22 = *A. bifasciatus*; 23 = *A. fulloi*; 24~26 = *A. japonicus*; 27~28 = *A. orientalis*;.

COI	1	2	3	4	5	6	7	8	9	10	11	12	13	14	15	16	17	18	19	20	21	22	23	24	25	26	27
1																											
2	0.015																										
3	0.134	0.136																									
4	0.122	0.124	0.011																								
5	0.136	0.132	0.149	0.139																							
6	0.124	0.126	0.136	0.126	0.055																						
7	0.118	0.120	0.130	0.120	0.048	0.008																					
8	0.130	0.128	0.143	0.134	0.075	0.078	0.074																				
9	0.128	0.130	0.143	0.134	0.073	0.076	0.071	0.031																			
10	0.124	0.126	0.147	0.138	0.068	0.080	0.074	0.030	0.011																		
11	0.128	0.124	0.145	0.136	0.066	0.076	0.071	0.025	0.018	0.013																	
12	0.134	0.136	0.167	0.157	0.096	0.091	0.091	0.106	0.106	0.100	0.102																
13	0.134	0.130	0.141	0.132	0.134	0.128	0.122	0.139	0.143	0.132	0.134	0.143															
14	0.093	0.082	0.143	0.134	0.124	0.134	0.126	0.130	0.124	0.122	0.122	0.149	0.134														
15	0.098	0.087	0.147	0.138	0.128	0.136	0.132	0.136	0.126	0.128	0.128	0.149	0.136	0.016													
16	0.098	0.087	0.147	0.138	0.128	0.136	0.132	0.136	0.126	0.128	0.128	0.149	0.136	0.016	0.000												
17	0.100	0.095	0.157	0.147	0.136	0.126	0.124	0.130	0.126	0.124	0.122	0.144	0.137	0.036	0.037	0.037											
18	0.105	0.109	0.136	0.126	0.116	0.103	0.103	0.117	0.120	0.113	0.113	0.118	0.105	0.118	0.122	0.122	0.124										
19	0.109	0.111	0.153	0.143	0.141	0.132	0.128	0.144	0.134	0.138	0.142	0.143	0.117	0.124	0.126	0.126	0.124	0.098									
20	0.128	0.128	0.151	0.137	0.143	0.143	0.139	0.140	0.132	0.130	0.132	0.151	0.130	0.122	0.126	0.126	0.132	0.120	0.130								
21	0.128	0.128	0.151	0.137	0.143	0.143	0.139	0.140	0.132	0.130	0.132	0.151	0.130	0.122	0.126	0.126	0.132	0.120	0.130	0.000							
22	0.128	0.128	0.151	0.137	0.143	0.143	0.139	0.140	0.132	0.130	0.132	0.151	0.130	0.122	0.126	0.126	0.132	0.120	0.130	0.000	0.000						
23	0.080	0.080	0.141	0.132	0.118	0.109	0.105	0.122	0.124	0.122	0.120	0.124	0.107	0.106	0.108	0.108	0.102	0.098	0.096	0.118	0.118	0.118					
24	0.089	0.087	0.153	0.143	0.134	0.136	0.130	0.136	0.132	0.130	0.128	0.149	0.117	0.096	0.098	0.098	0.110	0.109	0.111	0.113	0.113	0.113	0.087				
25	0.089	0.091	0.155	0.145	0.134	0.130	0.124	0.134	0.128	0.126	0.128	0.147	0.117	0.096	0.098	0.098	0.104	0.105	0.113	0.117	0.117	0.117	0.086	0.006			

Table S1. Pairwise genetic distances for *COI* gene sequences of *Anastatus*.1~2 = *A. taibaiensis* n. sp.; 3~4 = *A. caeruleus* n. sp.; 5~7 = *A. garygibsoni* n. sp.; 8~11 = *A. formosanus*.; 12 = *A. dexingensis*; 13 = *A. meilingensis*; 14~17 = *A. gastropachae*; 18 = *A. shichengensis*; 19 = *A. gansuensis*; 20~22 = *A. bifasciatus*; 23 = *A. fulloi*; 24~26 = *A. japonicus*; 27~28 = *A. orientalis*;.

26	0.086	0.089	0.153	0.143	0.122	0.126	0.120	0.126	0.124	0.115	0.120	0.143	0.119	0.098	0.104	0.104	0.106	0.103	0.115	0.117	0.117	0.117	0.086	0.016	0.016		
27	0.105	0.107	0.141	0.132	0.132	0.130	0.128	0.122	0.111	0.109	0.117	0.143	0.109	0.111	0.113	0.113	0.117	0.111	0.105	0.139	0.139	0.139	0.087	0.104	0.102	0.098	
28	0.105	0.107	0.139	0.130	0.130	0.128	0.126	0.120	0.109	0.107	0.115	0.141	0.109	0.109	0.111	0.111	0.119	0.107	0.100	0.134	0.134	0.134	0.085	0.105	0.104	0.096	0.005
